# Interpopulation differences in expression of candidate genes for salinity tolerance in winter migrating anadromous brown trout (*Salmo trutta *L.)

**DOI:** 10.1186/1471-2156-9-12

**Published:** 2008-01-29

**Authors:** Peter F Larsen, Einar E Nielsen, Anders Koed, Dennis S Thomsen, Pål A Olsvik, Volker Loeschcke

**Affiliations:** 1 Technical University of Denmark, Danish Institute for Fisheries Research, Department of Inland Fisheries, DK-8600 Silkeborg, Denmark; 2 Svendborg County, Svendborgvej 135, DK-5762 Vester Skerninge, Denmark; 3 University of Aarhus, Department of Biological Sciences, DK-8000 Aarhus C, Denmark; 4 National Institute of Nutrition and Seafood Research, PO Box 2029 Nordnes, N-5817 Bergen, Norway

## Abstract

**Background:**

Winter migration of immature brown trout (*Salmo trutta*) into freshwater rivers has been hypothesized to result from physiologically stressful combinations of high salinity and low temperature in the sea.

**Results:**

We sampled brown trout from two Danish populations entering different saline conditions and quantified expression of the *hsp70 *and *Na/K-ATPases α 1b *genes following acclimation to freshwater and full-strength seawater at 2°C and 10°C. An interaction effect of low temperature and high salinity on expression of both *hsp70 *and *Na/K-ATPase α 1b *was found in trout from the river entering high saline conditions, while a temperature independent up-regulation of both genes in full-strength seawater was found for trout entering marine conditions with lower salinities.

**Conclusion:**

Overall our results support the hypothesis that physiologically stressful conditions in the sea drive sea-run brown trout into freshwater rivers in winter. However, our results also demonstrate intra-specific differences in expression of important stress and osmoregulative genes most likely reflecting adaptive differences between trout populations on a regional scale, thus strongly suggesting local adaptations driven by the local marine environment.

## Background

Most fish species migrate at some stage during their life and migration takes place on a variety of temporal and spatial scales. Generally migration is related to feeding or reproduction [1-3], however, in some situations the reasons for migration are largely unknown.

Anadromous salmonids are renowned for their migration between freshwater spawning and nursery areas and marine feeding habitats [4,5]. The flexibility of such life-history related migration varies greatly among species ranging from completely fixed anadromous, e.g. pink salmon (*Oncorhynchus gorbuscha*) to entirely non-anadromous/resident life histories such as the Mexican golden trout (*Oncorhynchus chrysogaster*) [6]. For other species extreme flexibility can be found with anadromous and resident individuals within the same population, e.g. Atlantic salmon (*Salmo salar*), brook char (*Salvelinus fontinalis*), Arctic char (*Salvelinus alpinus*), brown trout (*Salmo trutta*), sockeye salmon (*Oncorhynchus nerka*) and rainbow trout (*Oncorhynchus mykiss*) (reviewed by [7]). The brown trout is well-known for its highly flexible life-cycle [8,9]. The anadromous form of brown trout, known as sea-trout, enter the marine environment on feeding migration as juveniles at the age of 1–3 years and they return to freshwater to spawn as mature adults after 0.5–3 years in the sea [10,11]. However, a proportion of the sea population re-enters freshwater before reaching maturity without any apparent explanation. This migration is commonly undertaken during winter and therefore it has been hypothesized as a strategy to escape the physiologically very stressful combination of low temperature and high salinity in the sea. Previous studies have indeed shown that physiological suboptimal temperatures and salinities can critically lower osmoregulation ability in sea-run brown trout [12-14].

Since anadromous brown trout populations may experience different local combinations of salinity and temperature during their sea phase the ability of responding to osmoregulatory stress may vary among populations. As a result they may have developed different osmoregulatory abilities/adaptations to local physical and chemical environmental conditions. Such local adaptations are defined as when individuals in their native environment on average have higher fitness than individuals/genotypes originating from a population inhabiting another environment [15]. So far, no studies have assessed to what extent variation in osmoregulatory capacities may be related to adaptation to the local marine environment in anadromous salmonids. However, some studies have suggested that the local marine environment could constitute an important component in relation to local adaptation in brown trout, e.g. Hansen *et al*. (2002). They showed that the major requirements for local adaptation to evolve in Danish brown trout populations were fulfilled, i.e. sufficient spatial genetic differentiation [16,17], temporal stability of genetic composition and large effective population sizes. Furthermore they demonstrated that adaptation most likely would occur on a regional basis encompassing several populations encountering similar marine selection regimes [16]. Finally, tagging experiments suggest that many populations of sea-run brown trout from different regions experience different environmental challenges in the sea. Some populations have been shown to have very local migration patterns close to the river of origin, while fish from other populations undertake long and different feeding migrations [18].

**Figure 1:**
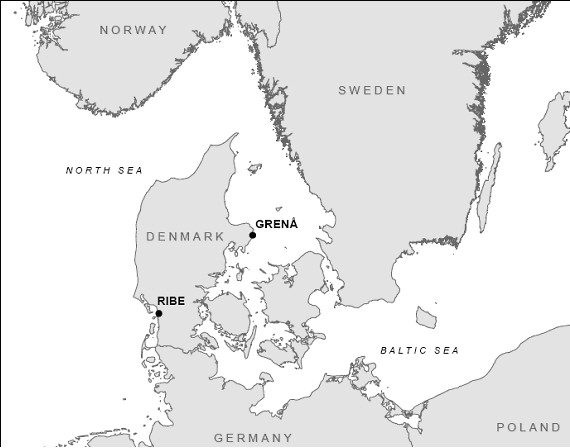
Map showing the location of River Ribe and River Grenaa in Denmark, represented by the towns of same name where the rivers enter the sea.

In this study the two trout populations originate from rivers draining into sea areas with highly different salinity conditions. The first population (River Ribe) enters full strength seawater in the North Sea/Wadden Sea (33 ppt) and the second population (River Grenaa) enters brackish water in the Kattegat (24 ppt) (Figure 1). Hansen *et al*. (2002) showed genetic structuring on a regional scale among Danish brown trout populations potentially related to sea salinity. Furthermore unpublished data from our lab has demonstrated significant (P < 0.0001) genetic differentiation between trout from river Grenaa and river Ribe with an F_ST _for seven microsatellite loci of 0.045 (95% confidence intervals bootstrapped across loci = 0.017 – 0.080).

Changes in gene expression has been suggested as an important component for adaptation to different environmental conditions [19-22]. However, so far, no studies are available where variation in gene expression related to osmoregulatory capacities under salinity and temperature stress in natural fish populations have been studied. Thus, Fangue *et al. *(2006) studied intraspecific variation in expression of a heat shock gene, *hsp70*, in relation to thermal tolerance in two populations of killifish (*Fundulus heteroclitus*) distributed along a steep thermal gradient on the East-coast of North America: They showed a tight coupling of thermal tolerance to the heat shock response and a higher expression of *hsp70*, following heat stress in northern compared to southern killifishes, most likely because southern fishes had adapted to higher annual temperatures [23]. Therefore, studying the pattern and variation in gene expression among natural populations shows high potential for revealing important functional differences involved in local adaptation to different environmental conditions.

Heat shock proteins (hsps) are good candidate genes for studying physiological stress since they are induced by multiple stressors, including osmotic and temperature stress. They are known to posses cytoprotective functions working as highly effective molecular chaperons refolding or degrading misfolded proteins [24]. Especially *hsp70 *is a commonly used indicator of physiological stress in multiple organisms, including fishes [25-27]. In relation to osmotic stress previous studies have suggested that hsps are important elements for acclimation of Atlantic salmon (*Salmo salar*) to hyperosmotic conditions [28] but also to restore osmotic homeostasis following osmotic stress [25]. Another group of interesting candidate genes for osmotic stress are the Na/K-ATPase genes. Na/K-ATPases are known to be critical for maintenance of ion balance and homeostasis of teleost fishes [29] and especially migration from seawater to freshwater, and vice versa, requires large changes in levels of Na/K-ATPase proteins and activity [4]. A number of recent studies have demonstrated a tight correlation between expression of Na/K-ATPase gene expression and environmental salinity in several salmonids, including brown trout [30-32].

In this study we investigated intra- and interpopulation variation in gene expression of *hsp70 *and the *Na/K-ATPase α 1b *subunit in winter migrating anadromous brown trout following acclimation to different combinations of salinity and temperature stress in a "common garden" setup. We wanted to study whether low temperatures at sea in winter could result in a stressful interaction effect explaining migration of sea trout into freshwater rivers. Secondly, we wanted to test the hypothesis of intraspecific variation in gene expression and salinity tolerance related to the local marine conditions experienced by trout from rivers entering different marine conditions. The results are discussed in relation to population structure, local adaptation and conservation of anadromous salmonids, in particular in relation to transplantation and reestablishment of brown trout populations.

## Results

**Figure 2:**
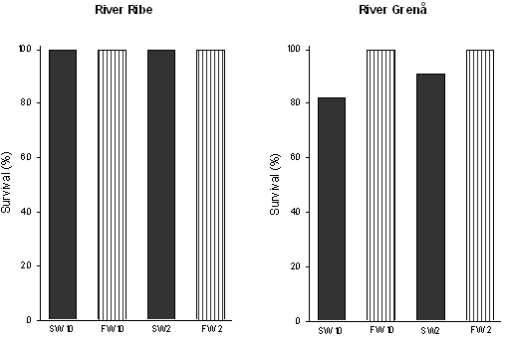
Survival of trout from River Ribe and River Grenaa following acclimation to freshwater (FW) and seawater (SW) at 2°C and 10°C (number of individuals in experimental groups: n = 9–11).

No mortality was observed in trout from River Ribe at any salinity or temperature (Fig. 2). In trout from River Grenaa no mortality was observed in freshwater, but in seawater a mortality of 18% and 9% was observed at 10°C and 2°C, respectively (Fig. 2).

**Figure 3:**
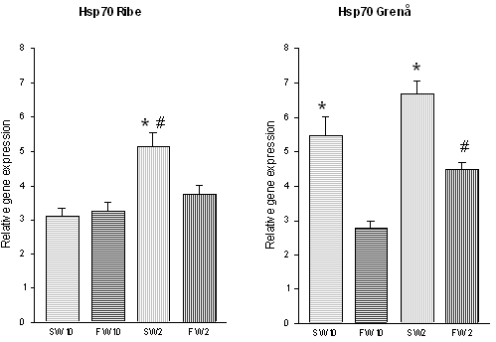
Expression of *hsp70 *in gill tissue of trout from River Ribe and River Grenaa following acclimation to freshwater (FW) and seawater (SW) at 2°C and 10°C. Amounts of *hsp70 *mRNA are normalized to the corresponding EF1α abundance from the same sample, and mean values are expressed in arbitrary units ± SE (number of individuals in experimental groups: n = 8). * indicates significant difference between salinity treatments (P < 0.05) and # indicates significant difference between temperature acclimation groups at the same salinity (P < 0.05).

Induced levels of *hsp70 *were found to be significantly higher in trout from River Ribe acclimated in seawater and 2°C compared to all other Ribe samples (Fig. 3). Furthermore, an interaction effect of both salinity (p < 0.05) and temperature (p < 0.01) was observed in expression of *hsp70 *in trout from River Ribe (p < 0.01). In trout from River Grenaa significantly higher levels of *hsp70 *was observed in trout from seawater at both 10°C and 2°C (Fig. 3) compared to freshwater acclimated trout. Moreover we found *hsp70 *to be significantly higher expressed in trout from freshwater at 2°C compared to 10°C (Fig. 3). The two-way ANOVA showed a significant effect of both salinity (p < 0.01) and temperature (p < 0.01), but no interaction effect was observed (p = 0.5314) in River Grenaa trout. A comparison of intra-specific variation, showed a significant higher expression of *hsp70 *in trout from River Grenaa in seawater at both 2°C (p < 0.05) and 10°C (p < 0.01) compared to trout from River Ribe. No significant differences in expression of *hsp70 *were observed in freshwater.

**Figure 4:**
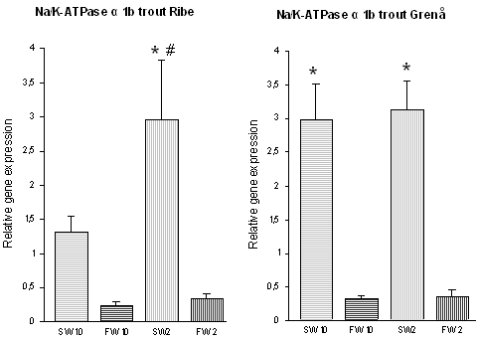
Expression of *Na/K-ATPase α 1b *in gill tissue of trout from River Ribe and River Grenaa following acclimation to freshwater (FW) and seawater (SW) at 2°C and 10°C. Amounts of *Na/K-ATPase α 1b *mRNA are normalized to the corresponding EF1α abundance from the same sample, and mean values are expressed in arbitrary units ± SE (number of individuals in experimental groups n = 8). * indicates significant difference between salinity treatments (P < 0.05) and # indicates significant difference between temperature acclimation groups at the same salinity (P < 0.05).

In general, the *Na/K-ATPase α 1b *gene was observed to be significantly higher expressed in seawater compared to freshwater in trout from both populations, although this difference was not significant in trout from River Ribe at 10°C (Fig. 4). The two-way ANOVA identified an interaction effect of temperature (p < 0.05) and salinity (p < 0.01) in trout from River Ribe (p < 0.05). In trout from River Grenaa a highly significant effect of salinity (p < 0.01), but no temperature (p = 0.7407) nor interaction effect was observed (p = 0.8503) in expression of the *Na/K-ATPase α 1b *gene. In the intra-specific comparison, trout from River Grenaa showed a significantly higher expression of *Na/K-ATPase α 1b *in seawater at 10°C (p < 0.01) compared to trout from River Ribe at the same temperature (Fig. 4), whereas no significant difference was observed at 2°C (p = 0.2738). No significant differences in expression of *Na/K-ATPase α 1b *were observed in freshwater.

## Discussion

In this study we identified a strong interaction effect of low seawater temperature and expression of *hsp70 *and *Na/K-ATPase α 1b *in sea trout from River Ribe, thereby supporting previous suggestions that sea-run brown trout enter freshwater rivers in winter due to osmoregulatory stress in relation to the combination of high salinity and low temperatures. At the same time we observed strong indications of lower salinity tolerance in trout from River Grenaa (seawater mortality); combined with higher expression of *hsp70 *and *Na/K-ATPase α 1b *in trout from River Grenaa maintained in full strength seawater demonstrating intraspecific variation in gene expression and osmoregulative capacities between the two trout populations. This variation most likely reflects adaptive differences between populations on a regional scale and strongly suggests adaptation to the local marine environment.

### Freshwater migration of sea-run brown trout

Winter migration of sea-run brown trout into freshwater rivers has been suggested as a strategy to overcome physiological stress resulting from the combination of high salinity and low temperature in the marine environment potentially resulting in lethal conditions for brown trout [5,14]. In this study, we demonstrated a significant interaction effect of "cold" seawater and the expression of the general stress gene *hsp70 *in River Ribe trout, whereas in trout from River Grenaa we observed significant *hsp70 *induction in both seawater experiments independent of temperature. These observations indicate physiological stress for trout in high saline waters, particularly in winter where the general metabolism is low. Similarly to expression of *hsp70 *we observed an interaction effect of cold seawater and expression of the *Na/K-ATPase α 1b *gene in trout from River Ribe and a highly significant up-regulation of the *Na/K-ATPase α 1b *gene in trout from River Grenaa following seawater acclimation. Combined, the results presented here show that the physiological stressful combination of low temperature and seawater salinity could be responsible for "triggering" migration into freshwater in sea-run brown trout in winter. This observation is in concordance with previous suggestions that some salmonid species may not be fully able to compensate for a reduction in seawater temperature by increasing gill Na/K-ATPase expression and instead induction of heat shock proteins may be highly important for cyto-protection under cold hypersaline conditions [28].

### Intra-specific variation in gene expression

Most studies focusing on osmo-regulation in salmonid fishes have aimed at describing the genes involved [33] and direction of gene expression [4] in comparative studies among species. Few studies have investigated the intra-specific variation in gene expression, and so far no studies have demonstrated any significant intraspecific differences that may explain local adaptive differences in relation to osmoregulative capacities [32,34]. Therefore, the present study is the first to document intra-specific differences in gene expression in a salmonid fish species.

We have shown a higher seawater tolerance and lower expression of *hsp70 *and *Na/K-ATPase α 1b *in trout from River Ribe strongly indicating higher fitness of River Ribe trout in full-strength seawater. Following the definition of Kawecki and Ebert (2004) local adaptations can be defined as when individuals have a higher fitness in their local environment compared to individuals from a foreign population in the same environment [15]. According to this definition our results strongly suggest local adaptation in trout from River Ribe because they apparently have higher fitness in their native high saline environment than trout from River Grenaa. The difference between the two populations is most likely caused by the difference in the marine habitats experienced by the two trout populations. Trout from River Grenaa naturally enter brackish water in the Kattegat Sea, whereas trout from River Ribe enter full-strength seawater, thus explaining why trout from River Grenaa are generally more stressed under high saline conditions. Consequently our results suggest that intra-specific variation in gene expression in relation to salinity tolerance might be important for maintaining population structure among Danish brown trout populations entering different marine conditions. These speculations are supported by Hansen *et al. *(2002) who suggested that trout populations entering different marine environments may be differently adapted on a regional scale based on seawater salinity.

### Sampling in nature

Several factors concerning sampling of fishes in nature for experimental gene expression studies might potentially have affected the results in the present study. Multiple non-genetic environmental factors affecting gene regulation have previously been discussed [35], however studying environmental stress other factors such as pre-treatment or "hardening" might also affect results [36]. This could potentially arise from that trout from River Ribe have spent a part of their life in full-strength seawater in the North Sea, and therefore, when they are acclimated to seawater in this experiment they may not respond so drastically to cold seawater compared to trout from River Grenaa, that, most likely, have fed in the more brackish Kattegat Sea. Moreover, studying migrating salmonids it is generally known that they may "lose" their "seawater regulative capacity" following long-term acclimation to freshwater [37]. However, this ability is known to be regained following acclimation to full strength seawater within 10–15 days – estimated for Atlantic salmon [4] and rainbow trout [38]. Therefore in this study we ensured the seawater acclimation to go on for 19 days in order to make sure potential effects of "time in freshwater" are eliminated. In addition, trout were caught as immature trout, at the same size (no significant differences in length) and they were treated in the same way, kept under the same laboratory conditions, and we therefore consider it unlikely that non-genetic factors have had a major impact on gene expression results in this study. Finally, it is important to consider that we only sampled sea trout in freshwater and therefore our explanation on what might "trigger" migration into freshwater only accounts for this group of trout. Especially since it is well-known that multiple life-history strategies exist in brown trout [12] it is unclear to what extent winter migration into freshwater or extended marine stay is dependent on life-history or triggered by specific environmental conditions. Therefore, it is possible that other trout from the same populations, or from other closely located populations, may over-winter in the sea and that such trout may have genetic adaptations and/or physiological advances doing so.

### Conservation

The observation of increased mortality in eastern Danish sea trout acclimated to full-strength seawater might explain previous observations of increased mortality of stocked hatchery trout compared to indigenous trout in Denmark, e.g. Ruzzante *et al. *(2004). These authors found, despite extensive stocking for several consecutive years, that for brown trout almost none of the returning spawning individuals were of hatchery origin and therefore suggested that stocked anadromous trout had a very high mortality at sea [17]. In a similar study by Hansen *et al. *(2000), they showed that stocked trout had a higher survival and reproductive success as resident trout than as anadromous trout [39]. The low survival of stocked trout entering the sea in these two studies may potentially result from that the majority of hatchery reared brown trout stocked in Denmark during the last forty years originate from the Hårkær hatchery. This hatchery was historically founded with wild trout caught in River Kolding in south-eastern Denmark [8]. We therefore hypothesize that stocked trout from the hatchery strain entering seawater as anadromous individuals could experience high osmotic stress and seawater mortality, in a similar way as the trout from River Grenaa in this present experiment. This might, to some extent, explain the extremely low survival of stocked anadromous trout previously observed [16,17]. However, the Hårkjær hatchery has been operating for more than one hundred years, and we can therefore not rule out whether hatchery induced selection e.g. to strictly freshwater or potential human induced selection regimes may have resulted in a lower fitness of hatchery trout in the wild. However, we do consider it likely that adaptive differences in salinity tolerance and gene expression among trout populations may play an important role for fitness of anadromous trout, both in natural populations and in artificial breed hatchery strains used for stocking activities.

## Conclusion

Our results support the hypothesis that physiologically stressful conditions in the sea drive sea-run brown trout into freshwater rivers in cold winters. Furthermore, our results also demonstrate intra-specific differences in expression of important stress and osmoregulative genes most likely reflecting adaptive differences between trout populations on a regional scale. The observation that intra-specific variation in salinity tolerance and gene expression might explain previous non-successful stocking activities support the ongoing conservation strategy for trout in Denmark. At present the target is to increase natural reproduction of indigenous fish in their native rivers, thereby maintaining local adaptations. If very poor or completely missing recruitment is the case supportive breeding using fry from wild caught spawners is allowed. In the case that the indigenous population has been extirpated reintroduction is conducted using fish from a healthy nearby population experiencing similar environmental conditions both in freshwater and in the sea. Potentially, due to the large differences in salinity tolerance and expression of osmoregulative genes between the two trout populations studied here, several major problems could arise as a result of interbreeding/hybridization between indigenous and stocked trout – e.g. degradation of local adaptations through breakdown of co-adapted gene complexes [40].

## Methods

### Sampling and experimental setup

Immature sea trout were caught by electrofishing in the lower parts of the River Grenaa (n = 39) and the River Ribe (n = 43) in January and February 2005. Trout of similar sizes, mean fork length from River Grenaa (33.2 cm) and River Ribe (35.7 cm), were transported to the Danish Institute for Fisheries Research, Department of Inland Fisheries, PIT-tagged and acclimated to 10°C in re-circulated freshwater. Trout from each of the two rivers were split into two groups maintained at 10°C in freshwater and 10°C in seawater, respectively. Within these groups (freshwater and seawater) trout were again divided into two groups where the water temperature was lowered from 10°C to 2°C over a period of four days. Sampling of gill tissue for this experiment was conducted 10 days after lowering the temperature from 10°C to 2°C, which was 19 days from the onset of the acclimation to seawater.

The gill is known as the main organ for osmo-regulation in fishes [14,41] and therefore we focused on this specific tissue. Dissected gill tissue was immediately placed in RNAlater following the manufacturer's instructions (Qiagen, Hilden, Germany). RNAlater preserved samples were stored overnight at 4°C and then stored at -20°C until RNA extraction. All trout were maintained at a 10 h light/14 h dark cycle and no food was supplied during the experiment. Thus, since all fish were sampled on the same day they were also fasted for the same period of time.

### RNA extraction

Total RNA was extracted using both the Trizol method (Invitrogen, Carlsbad, CA, USA) and the RNeasy minikit (Qiagen, Hilden, Germany). Extractions were performed as recommended by the manufacturers except for an additional DNAse treatment step for 15 min at 25°C in order to remove any remaining genomic DNA in the RNeasy extracted samples (Qiagen, Hilden, Germany). Total RNA was stored at -20°C. Concentration of extracted RNA was determined at 260 nm in a standard Hellma cuvette (path length 10 mm) using the GeneQuant II (RNA/DNA Calculator, Pharmacia Biotech). Total RNA quality was routinely analyzed using 2% agarose gel electrophoresis.

### Reverse transcription of RNA and real-time quantitative PCR (qRT-PCR)

Reverse transcription of total RNA into cDNA was performed using the SuperScriptII RNase H-Reverse Transcriptase kit (Invitrogen, Carlsbad, CA, USA) in a reaction volume of 20 μl containing 1× reaction buffer, 5 mM MgCl_2_, 1 mM dNTP mixture, 0.3 μl of anchored oligo-(dT)_20 _primer (2.5 μg/μl), 0.9 μl of SuperScriptII reverse transcriptase, and 1 μg of total RNA. Following, samples were diluted ten fold and stored at -20°C until qRT-PCR analysis.

For the Real-time quantitative PCR (qRT-PCR) analysis previously published primer pairs were used for *Na/K-ATPase α 1b*-subunit [38] and *hsp70 *[42]. Results were normalized to expression of elongation factor 1α [EMBL: AF321836], using the following primers: EF-1α forward: 5'-GAG AAC CAT TGA GAA GTT CGA GAA G-3'; EF-1α reverse 5'-GCA CCC AGG CAT ACT TGA AAG-3'. This house keeping gene has been used and found suitable and stable for gene expression normalization (e.g. [21-23]).

PCR products from all primer pairs were checked on a 2% agarose gel to verify primer specificity and PCR product length and to ensure that they only produced a single amplicon when trout cDNA served as template. Furthermore dilution series were performed to verify amplification efficiency of primer pairs [43].

qRT-PCR was performed on the Ligthcycler 1.2 (Roche Diagnostics, Mannheim, Germany) using SYBR Green chemistry (LightCycler FastStart DNA Master SYBR Green I kit, Roche) and the Lightcycler relative quantification software 3.5 (Roche Diagnostics, Mannheim, Germany). Using this software we employed the fully automated method for C_T_-determination called the "*2^nd ^Derivative Maximum*" calculation. The C_T_-value is the cycle where the 2^nd ^derivative is at its maximum and ideally this should always be in the heart of the log-linear portion of the reaction. Moreover this method is more objective and reproducible than human mediated crossing point settings (Lightcycler relative quantification software manual). All qRT-PCR reactions were performed as follows: 10 min at 95°C, followed by 40 cycles of 95°C for 15 s, 60°C for 10 s and 72°C for 10 s. Melting curve analysis was performed following each reaction to confirm the presence of only a single product in the reaction. Negative control reactions were performed for all samples using RNA that had not been reverse transcribed to control for the possible presence of genomic DNA contamination. Genomic DNA contamination was present in all samples, but never constituted more than 1:5000 of starting cDNA copy numbers (data not shown). Therefore genomic DNA contamination reflects only a minor fraction of the final PCR-product and only an insignificant source of error for the data analysis.

### Statistical Analysis

Relative gene expression values were calculated, using the "Comparative method", according to [44]. Data are presented as mean expression levels ± SE. Gene expression levels were compared using one-way ANOVA, and multiple pairwise comparisons were conducted using Tukeys test between salinities (within populations) or between populations (maintained at the same salinity). Finally, two-way ANOVA was conducted to test for an interaction effect of salinity and temperature within the two trout populations. Significance level was set at α = 0.05 in all analyses and analyses were conducted using the PAST statistics software [45].

## Authors' contributions

PFL, EEN, AK and DST designed the experiment and PFL carried out the molecular and statistical analyses, DST did the sampling of trout. PAO supplied primers for the hsp70 assay and together with VL provided valuable technical input to the data interpretation. All authors read and approved the final manuscript.
